# Transmission of recombinant enterovirus A76 (EV-A76) in Xinjiang Uighur autonomous region of China

**DOI:** 10.1080/22221751.2022.2149350

**Published:** 2022-12-20

**Authors:** Xiaolei Li, Huanhuan Lu, Qiang Sun, Peng Zheng, Bo Zhang, Hui Cui, Haishu Tang, Hehe Zhao, Ying Liu, Jie Jiang, Jinbo Xiao, Yong Zhang

**Affiliations:** a WHO WPRO Regional Polio Reference Laboratory, National Health Commission Key Laboratory for Biosafety, National Institute for Viral Disease Control and Prevention, Chinese Center for Disease Control and Prevention, Beijing, People’s Republic of China; b Jinan Institute for Food and Drug Control, Jinan, People’s Republic of China; c Teaching Department of Basic Medicine, Taishan Vocational College of Nursing, Tai’an, People’s Republic of China; d Xinjiang Uygur Autonomous Region Center for Disease Control and Prevention, Urumqi, People’s Republic of China; e Center for Biosafety Mega-Science, Chinese Academy of Sciences, Wuhan, People’s Republic of China

**Keywords:** Enterovirus A76, transmission, recombination

## Abstract

Enterovirus 76 (EV-A76) is a serotype of enterovirus A and has been rarely reported. In this paper, we present the genetic characteristics of 15 EV-A76 isolates reported to circulate in the Xinjiang Uighur autonomous region of China in 2011. Sequence analysis revealed that all Chinese EV-A76 isolates had high similarity (> 98.3%) in the *VP1* region, and five Chinese EV-A76 isolates were selected for whole genome sequencing based on *VP1* nucleotide divergence. Similarity plots and boot-scanning analyses revealed frequent intertypic recombination in the nonstructural region of the EV-A76 isolate, as found with the EV-A89 donor sequence (also isolated in Xinjiang). The breakpoint of recombination is around nucleotide 3960, and the recombinant fragments covered part *2C* and all *P3* regions. This study increases publicly available EV-A76 nucleotide sequence and further our understanding EV-A76 molecular epidemiology.

The genus *Enterovirus*, within family *Picornaviridae*, order *Picornavirales*, contains 15 species, among which four main species have been reported to cause human disease: Enterovirus A, B, C, and D (EV-A to EV-D). EV-A76 is a rare reported serotype within EV-A, and at present, only a few countries have reported cases or outbreaks of EV-A76, and only six complete genome sequences are available in the GenBank database (as of October 2022) [1]. The diversity of genetic variability in enteroviruses is mostly due to nucleotide substitutions and recombination [2].

An enterovirus surveillance programme was launched in southern Xinjiang in 2011 with the aim of evaluating enterovirus carriage among patients with acute flaccid paralysis (AFP) and their contacts [3]. A total of 1250 stool specimens were collected from 1021 children (229 AFP cases and 792 healthy contacts) and 372 enteroviruses were typed by viral isolation and molecular typing method, including 39 EV-A, 232 EV-B and 101 EV-C. Among EV-A, EV-A76 was the most numerous (15), followed by EV-A71 (10), EV-A90 (5), CVA14 (3), CVA16 (2), CVA2 (1), CVA4 (1), CVA8 (1), and EV-A89 (1). Fifteen EV-A76 (4.0% of positives) was detected in two AFP patients and 13 healthy contacts in the Hotan, Bortala Mongol, and Kashgar prefectures in this surveillance programme. In this study, the origin, evolution, and epidemic characteristics of EV-A76 were analysed.

This study did not involve human experimentation. All sample collection and experimental procedures were approved by the Second Ethics Review Committee of the National Institute for Viral Disease Control and Prevention and the Chinese Center for Disease Control and Prevention.

Stool samples were collected from AFP patients and their contacts during the enterovirus surveillance programme initiated in southern Xinjiang in 2011, and viruses were isolated from original stool specimens by propagation in human rhabdomyosarcoma (RD) and human laryngeal epidermoid carcinoma (HEp-2) cell lines according to the standard protocol [4].

Viral RNA was extracted from the viral isolates using a QIAamp Viral RNA Mini Kit (Qiagen, Valencia, CA, USA). RT–PCR was performed with Primscript One-Step RT–PCR Kit Ver. 2 (Takara, Dalian, China) according to the manufacturer’s instructions. The primer pair EV76-VP1S/EV76-VP1A (upstream primer EV76-VP1S, 5′–TGATCCAGTGGAGGACATGA-3′; downstream primer EV76-VP1A, 5′–GCTGGATCAAAGTTGGGGTA-3′) were used in PCR amplification to yield fragments of approximately 1.0 kb spanning *VP1*. The PCR products obtained were purified using the QIAquick Gel extraction kit (Qiagen), and the amplicons were bidirectionally sequenced using an ABI PRISM 3130 Genetic Analyzer (Applied Biosystems, Hitachi, Japan). The Enterovirus genotyping tool and BLAST server were used to identify Enterovirus type [5]. Sequence analysis revealed that 15 Xinjiang EV-A76 isolates had high nucleotide similarity (> 98.3%) in the *VP1* region.

Five Xinjiang EV-A76 isolates were selected for whole genome sequencing based on *VP1* nucleotide divergence. The complete genome sequence was amplified using the “primer-walking” strategy and overlapping segments representing the entire genome were amplified by one-step RT–PCR using specific primers (Supplementary Table 1). The 10 *VP1* sequences and five full-length genome sequences detected in this study have been deposited in GenBank under accession numbers ON646231-ON646245, ON646232, ON646239, ON646240, ON646243, and ON646245 are whole genome sequences.

The evolutionary characteristics of the EV-A76 strains described in this study were investigated using the Maximum Clade Credibility (MCC) tree and Bayesian skyline plot generated by BEAST software (version 2.5) [6]. The nucleotide substitution model of HKY + G + I was strongly verified using jModelTest. The sequences in the dataset were marked with the isolation time to promote the calibration of the molecular clock. The dataset was first tested with appropriate parameters by comparing the best marginal likelihood estimation results with three different clock models and six different coalescent tree priors. After the pre-test, we adopted 80 million generations, and every 8000 generations were sampled. Tracer software (version 1.7.1) was used to check the convergent validity and effective sampling size of the parameters. The output trees were visualized using TreeAnnotator software (version 1.8.4) with a burn-in value of 1000, meaning every 10 percent of 10,000 sampled trees was exported. An analysis of the MCC tree revealed that the most recent common ancestor between Chinese and other isolates was 1987. After 1987, it gradually evolved into two branches (Figure 1(a)). A Bayesian skyline plot was also constructed using the settled model, and the analyses were performed using Tracer, the results illustrates that the effective population size remained unchanged until 2000 mainly because of the lack of sequence data. During the first phase (2001–2009), a slight downward trend was observed. Since 2010, the number of infected populations exhibited rapid growth within two years (Figure 1(b)).

**Figure 1.**
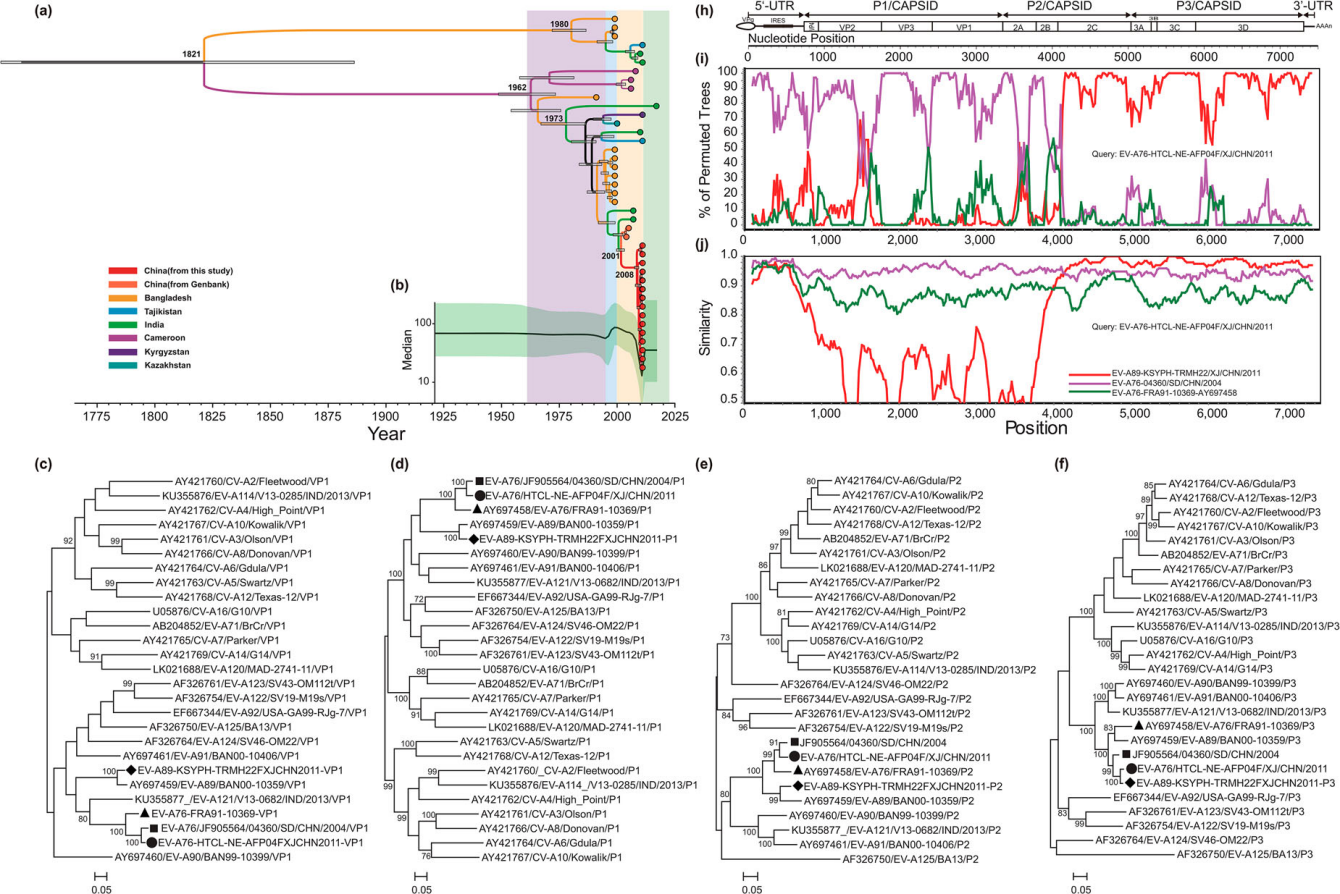
Phylogenetic analysis and recombination analysis of Xinjiang EV-A76 isolates. source: (a) The MCC phylogenetic tree and (b) the Bayesian Skyline Plot were constructed based on VP1 region of 41 sequences of EV-A76 strains worldwide. Phylogenetic tree based on the *VP1* (c), *P1* (d), *P2* (e), and *P3* (f) sequences of our EV-A76 isolate, the Shandong EV-A76 isolate, the Xinjiang EV-A89 isolate, and other EV-A prototype strains. (g) Gene structure organization, (h) similarity plot analyses of Xinjiang EV-A76 isolate, and (i) boot-scanning analysis of Xinjiang EV-A76 isolate.

Phylogenetic trees were constructed using the maximum likelihood method implemented in the MEGA software (version 11.0.11) [7] using the GTR + G model and 1000 bootstrap replicates. Bootstrap values greater than 70% were considered a support value. Phylogenetic trees were established, including an EV-A76 isolate in this study, an EV-A76 strain isolated in Shandong Province of China (04360/SD/CHN/2004), an EV-A89 strain isolated in Xinjiang in 2011 (EV-A89-KSYPH-TRMH22FXJCHN2011), and all EV-A protype strains in the GenBank database based on nucleotide sequences of *VP1*, *P1*, *P2,* and *P3* coding regions (Figure 1(c–f)). Both *VP1* and *P1* phylogenetic trees showed that the strain in this study clustered with the EV-A76 prototype and EV-A76 strain isolated in Shandong. However, in the phylogenetic trees based on *P3* coding regions, the EV-A76 isolate of this study not only converged with the EV-A76 strains but also clustered with the EV-A89 Xinjiang isolate, suggesting that Xinjiang EV-A76 isolates may have recombined with EV-A89 in the *P3* coding regions.

To further investigate the source of EV-A76 recombination, The nucleotide alignment containing the genome sequence of Xinjiang EV-A76 strains, EV-A76 prototype strain, and EV-A89 strain KSYPH-TRMH22F/XJ/CHN/2011 [8] was generated using the MEGA program (version 11.0.11). Once aligned, similarity plot and bootscaning analyses were performed using the Simplot program (version 3.5.1). And the results confirm the existence of recombination events between the EV-A76 strain in this study and the EV-A89 strain isolated in Xinjiang in the 2C and P3 regions (Figure 1(g–i)). The results indicated that all five Xinjiang EV-A76 sequences possessed the same recombination site. The breakpoint of recombination is around nucleotide 3960, and the recombinant fragments covered part *2C* and all *P3* regions of the genome, which is consistent with the results of the aforementioned phylogenetic trees. Thus, the co-circulation of various EV-A (e.g. EV-A76 and EV-A89) may be a key factor in facilitating the development of recombinant EV-A76.

In conclusion, 15 EV-A76 strains isolated from an outbreak in Xinjiang, China in 2011 were reported in this study, and sequence analysis showed that they belonged to the same origin. Five of these viruses were subjected to whole genome sequencing and bioinformatics analysis, and all of them were found to undergo intertypic recombination. This study increases publicly available EV-A76 nucleotide sequence, expands whole-genome sequences in GenBank, and provides further insight into the EV-A76 molecular epidemiology.

## Supplementary Material

Supplemental Material
