# Supplementary material for: Transmission of recombinant enterovirus A76 (EV-A76) in Xinjiang Uighur autonomous region of China
Source: Emerg Microbes Infect. 2022 Dec 20;12(1):2149350. doi: 10.1080/22221751.2022.2149350 (PMC9788713; doi:10.1080/22221751.2022.2149350)
Supplement: Supplemental Material [file TEMI_A_2149350_SM0388.docx]

**Supplementary Table 1. Primer sequences used in the “primer-walking” strategy for the whole genome sequencing of EV-A76**

| Primer name | Sequence (5’-3’) |
| --- | --- |
| 0001S48 | GGGGACAAGTTTGTACAAAAAAGCAGGCTTTAAAACAGCTCTGGGGTT |
| EV76-778S | GGGTCTCACGAGAACCAAAA |
| EV76-1008A | TACAGTCAATTGCGCGACTC |
| EV76-1639S | TCTGATGGAGCAACAACTGC |
| EV76-1838A | GGGATGTGAATTGCTGGAGT |
| EV76-2463S | TGATCCAGTGGAGGACATGA |
| EV76-2627A | GAAGTGGCTCCAGTTTCAGC |
| EV76-3085S | ACATTCGGTTCACACCCTGT |
| EV76-3302A | GCTGGATCAAAGTTGGGGTA |
| EV76-3832S | GCTTTTGGAACTGGCTTCAC |
| EV76-4014A | AAGCGCCAAGGTAGCAGTTA |
| EV76-4567S | CCACCAGACCCTGATCACTT |
| EV76-4770A | GCTCATGTTTGTGGAAGCAA |
| EV76-5230S | AATGTGGAAAAGCACCTTGG |
| EV76-5479A | AATGACCCTGGTCTGTCTGC |
| EV76-6062S | GAGTCAAAGAACCGGCAGTC |
| EV76-6292A | CCAGGTCAATTCCTTCCAGA |
| EV76-6953S | CTAGCTACCCCTTCCCCATC |
| EV76-7135A | GCATGGTTGGGTGAATAAGG |
| [7500A](https://www.baidu.com/link?url=g9zNl7MZwnx19UW2eutlXUr_F3KXzizfLNSeCH6o7so6fOcNlnJYIIuXQMvwwcAB7VXPIzC_45kjQ2kUwcFBS95UsJ-vCGl0JJ3c_g63SBp19kb62p4N7Z3S_sHGcxKT&wd=&eqid=efc8fae9000005970000000362ec7ff2) | GGGGACCACTTTGTACAAGAAAGCTGGG(T)_24_ |
